# *Arabidopsis* MSH1 mutation alters the epigenome and produces heritable changes in plant growth

**DOI:** 10.1038/ncomms7386

**Published:** 2015-02-27

**Authors:** Kamaldeep S. Virdi, John D. Laurie, Ying-Zhi Xu, Jiantao Yu, Mon-Ray Shao, Robersy Sanchez, Hardik Kundariya, Dong Wang, Jean-Jack M. Riethoven, Yashitola Wamboldt, Maria P. Arrieta-Montiel, Vikas Shedge, Sally A. Mackenzie

**Affiliations:** 1 School of Biological Sciences, University of Nebraska, Lincoln, Nebraska 68588, USA; 2 Department of Agronomy and Horticulture, University of Nebraska, Lincoln, Nebraska 68588, USA; 3 Department of Statistics, University of Nebraska, Lincoln, Nebraska 68588, USA; 4 Center for Biotechnology, University of Nebraska, Lincoln, Nebraska 68588, USA

## Abstract

Plant phenotypes respond to environmental change, an adaptive capacity that is at least partly transgenerational. However, epigenetic components of this interplay are difficult to measure. Depletion of the nuclear-encoded protein MSH1 causes dramatic and heritable changes in plant development, and here we show that crossing these altered plants with isogenic wild type produces epi-lines with heritable, enhanced growth vigour. Pericentromeric DNA hypermethylation occurs in a subset of *msh1* mutants, indicative of heightened transposon repression, while enhanced growth epi-lines show large chromosomal segments of differential CG methylation, reflecting genome-wide reprogramming. When seedlings are treated with 5-azacytidine, root growth of epi-lines is restored to wild-type levels, implicating hypermethylation in enhanced growth. Grafts of wild-type floral stems to mutant rosettes produce progeny with enhanced growth and altered CG methylation strikingly similar to epi-lines, indicating a mobile signal when *MSH1* is downregulated, and confirming the programmed nature of methylome and phenotype changes.

## Abstract (web summary)

Suppression of MutS HOMOLOGUE 1 (MSH1), a plant protein targeted to mitochondria and plastids, causes a variety of phenotypes. Here Virdi *et al.* show that MSH1 depletion in *Arabidopsis* results in heritable changes in nuclear DNA methylation, which can lead to enhanced growth vigour.

## Graphical abstract

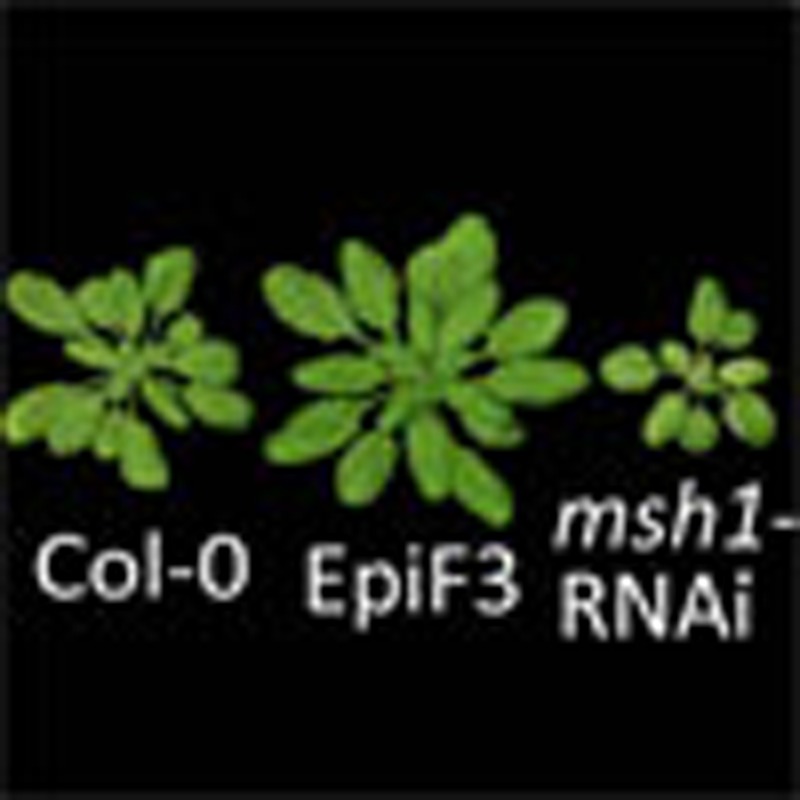

Evidence exists in support of a link between environmental conditions and epigenetic changes in plants and animals^1^,^2^,^3^,^4^. Transgenerational heritability of these changes remains a subject of investigation^5^,^6^, but studies in plants indicate that it is feasible to establish new and stable epigenetic states^7^,^8^,^9^. Here we demonstrate a link between plastid perturbation and nuclear epigenetic reprogramming through suppression of the nuclear-encoded *MutS HOMOLOGUE 1 (MSH1*) gene.

MSH1 is a mitochondrial- and plastid-targeting protein unique to plants and is involved in organelle genome stability^10^,^11^. In multiple plant species, variable phenotypes emerge when *MSH1* is lost or suppressed by RNA interference (RNAi)^1^,^11^. These phenotypes include leaf variegation, enhanced branching or tillering, dwarfing, delayed maturity and flowering, enhanced abiotic stress tolerance and, at least in *Arabidopsis*, a partial transition to perennial growth at short daylengths^1^. Generally, this range of phenotypes is observed in progeny of *msh1* mutants regardless of the parental phenotype, that is, both a phenotypically normal or aberrant *msh1* mutant plant can produce progeny displaying the full phenotypic range. This lack of direct heritability in *msh1* mutants implies a non-Mendelian mode of inheritance for the observed phenotypes. When induced by RNAi suppression, these phenotypes persist in a proportion of progeny, independent of the RNAi transgene^1^. Possible explanations for this behaviour include the creation of epigenetically derived phenotypes or perhaps the fixation of aberrant cytoplasm and associated phenotypes after *MSH1* restoration.

This study evaluates the evidence for heritable, epigenetic changes in association with MSH1 depletion in *Arabidopsis* and following genetic crossing of the *msh1* mutant to wild type. We show that MSH1 depletion leads to genome-wide methylation changes that may include non-CG context and largely within pericentromeric regions of the genome. Genetic crossing or grafting of the *msh1* mutant to wild type can lead to a dramatic enhancement of growth vigour. In this case, changes in the methylome predominantly influence distributed CG methylation. These observations are consistent with MSH1 as a novel component of the environmental sensing apparatus of the plant, perhaps serving to link the detection of environmental change to epigenetic response potential within the plant.

## Results

### Crossing of *msh1* leads to enhanced growth in *Arabidopsis*

**Figure 1:**
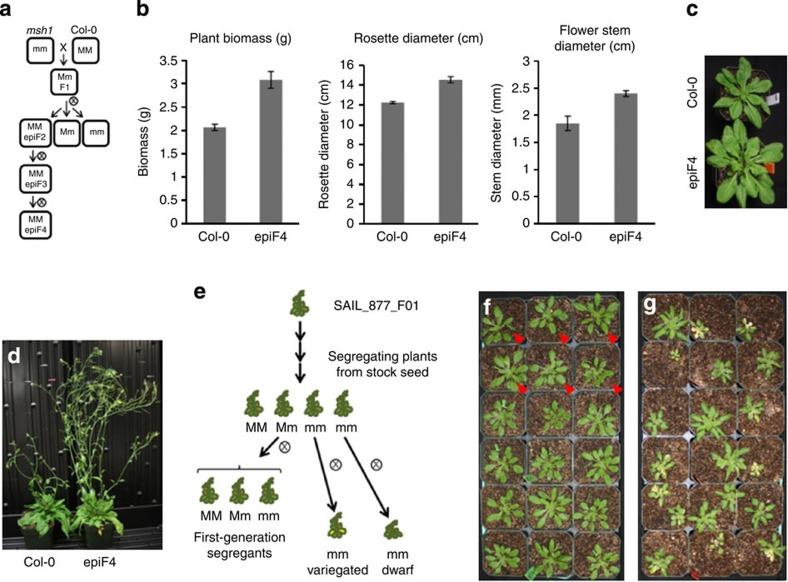
Phenotypically variable *msh1* mutants produce enhanced growth progeny on crossing to wild type. (**a**) Crossing scheme for creating epi-lines. (**b**) The epiF4 plants show enhanced plant biomass, rosette diameter and floral stem diameter relative to Col-0. (**c**) Enhanced growth phenotype of the epiF4; this enhanced growth is observed in ~100% of F4 progeny deriving from a selected epiF3 line. (**d**) The epiF4 phenotype at maturity. (**e**) Scheme to derive early-generation *msh1* materials for methylome analysis. (**f**) Segregating progeny from a single hemizygous plant. First-generation *msh1* −/− plants are marked with triangles. (**g**) Second-generation siblings from a single first-generation *msh1* −/− parent exhibited variegation and size variation.

In *Sorghum*, crossing *msh1* suppression lines to isogenic wild type restores normal growth and leads to enhanced vigour^12^. Restoration of normal progeny occurs regardless of the crossing direction, implying an epigenetic rather than a cytoplasmic cause. To investigate the possibility that similar phenotypes arise through epigenetic means and are heritable after restoration of *MSH1* in *Arabidopsis*, we carried out crossing experiments between *msh1* mutants and wild-type plants (Fig. 1). Crossing of wild-type Columbia-0 (Col-0) with a first-generation *msh1* mutant of *chm1-1*, which contains a point mutation^10^, resulted in increased phenotypic variation in the F2 progeny. By the F4 generation, markedly enhanced vigour was observed, with plants exhibiting larger rosettes and stem diameter with early flowering (Fig. 1), similar to observations in *Sorghum*^1^,^12^. Sequencing and alignment of the *chm1-1* genome produced no evidence of illegitimate recombination or rearrangement to account for the novel phenotypic variation, although there was evidence of previous introgressions from L*er* (Supplementary Fig. 1). In support of the crosses using *chm1-1*, *msh1* mutants were also obtained using an *msh1* T-DNA insertion mutant and RNAi. Hemizygous RNAi plants were self-pollinated to obtain RNAi-null (lacking transgene) progeny from which a dwarfed line was selected for crossing. Crossing of the T-DNA mutant and RNAi-derived dwarf plants to Col-0 likewise resulted in enhanced phenotypic variation in F2 lines (Supplementary Fig. 2).

### The MSH1 growth effect through perturbation of the plastid

Since the mutation of MSH1 affects both the mitochondria and plastids, but altered plant development in *Arabidopsis msh1* is conditioned by plastid changes^1^, we tested whether the enhanced growth vigour also emanated from plastid-associated effects. *Arabidopsis MSH1* hemicomplementation lines, derived by introducing a mitochondrial- versus chloroplast-targeted *MSH1* transgene to the *chm1-1* mutant^11^, distinguish mitochondrial and plastid contributions to the phenomenon. Plastid hemicomplementation lines (complemented for plastid effects but not mitochondrial) crossed as female to Col-0 resulted in a normal phenotype for some F_1_ progeny, but with 10 to 77% showing slow germination, leaf curling and delayed flowering. These altered phenotypes may be due to mitochondrial changes. In F1 progeny from crosses to the mitochondrial-complemented line, over 30% showed enhanced growth, larger rosette diameter and earlier flowering time, closely resembling F4 phenotypes from *chm1-1* × Col-0 (Supplementary Fig. 3a). These results, occurring independent of the transgene, were further confirmed in derived F_2_ populations (Supplementary Fig. 3b–f, Supplementary Table 1), indicating that *msh1*-deprived plastids are necessary for the growth vigour changes seen after crossing.

### Methylation changes with MSH1 disruption

To learn whether *msh1*-mediated growth changes were associated with epigenetic changes, we performed genome-wide bisulfite sequencing on material derived from early-generation *msh1* T-DNA mutants (Fig. 1e), thereby minimizing generational DNA methylation changes unrelated to MSH1 loss. A segregating Salk T-DNA line was obtained and a heterozygous individual was self-pollinated to yield *MSH1* +/+ (wild-type segregant), *MSH1* +/− heterozygotes and *msh1* −/− (considered first generation), each of which was included in bisulfite sequencing. Additional *msh1* −/− plants were self-pollinated to create second-generation *msh1* mutants, which recapitulated the variable phenotypes seen in *chm1-1*; individuals showing variegation (*msh1* gen2 variegated) and dwarfing (*msh1* gen2 dwarf) were included in bisulfite sequencing.

**Figure 2:**
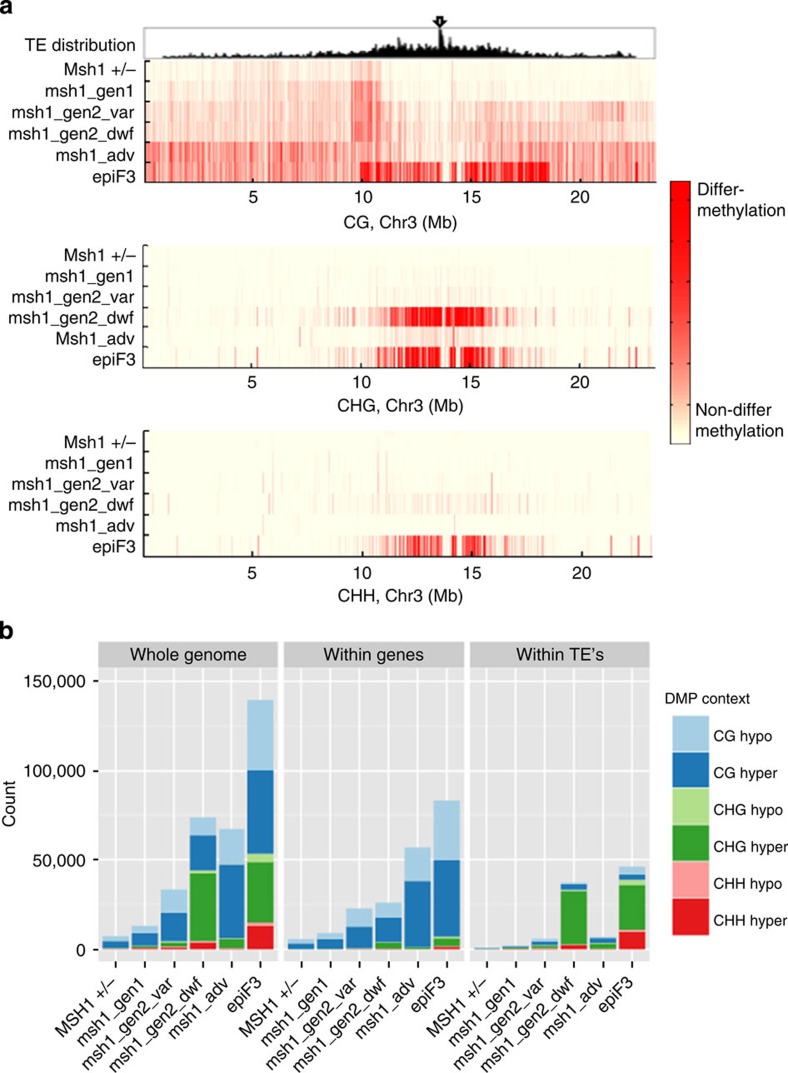
Pairwise DMP patterns of *MSH1* +/− and early *msh1* mutants when compared with wild-type segregants, and of *chm1-1* (msh1 adv) and epiF3 when compared with stock Col-0. (**a**) Distribution of CG, CHG and CHH-DMPs along chromosome 3. Top window, distribution of transposons; arrow indicates centromere. (**b**) Comparison of whole genome, gene and transposon pairwise DMP counts.

**Figure 3:**
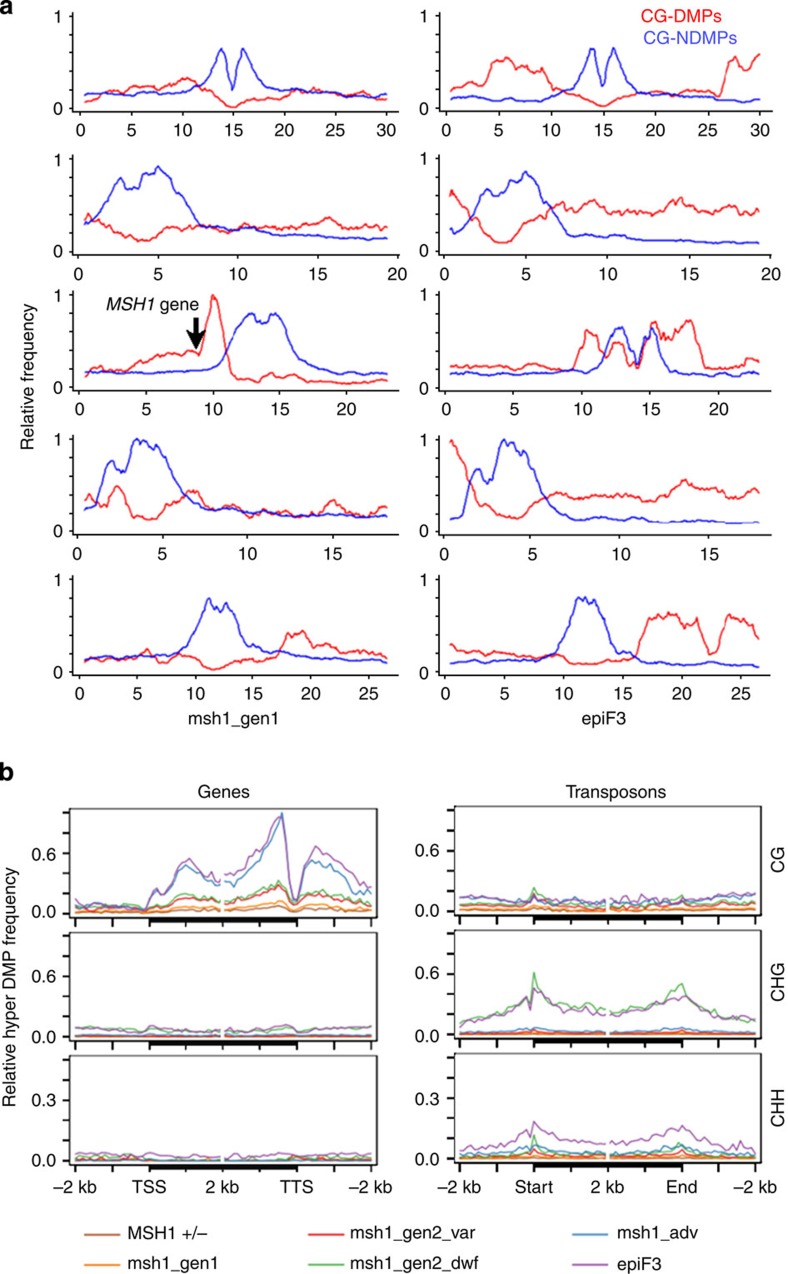
Unique patterning of DMPs in msh1 mutants and an enhanced growth epiF3 line. (**a**) Chromosomal distributions of pairwise CG-DMPs (red) and CG-NDMPs (blue) in a comparison of first-generation *msh1* to its segregating wild-type sibling (left), and epiF3 to stock Col-0 (all chromosomes normalized together in each comparison). Arrow indicates the position of the *MSH1* gene on chromosome 3. (**b**) Distribution of hypermethylated pairwise DMPs over genes and transposons.

Methylome analysis was first conducted based on pairwise comparisons of each *msh1* mutant to wild type. Generally, we observed increasing numbers of pairwise CG differentially methylated positions (DMPs) along chromosome arms (Fig. 2a), a proportion of which is likely still due to unavoidable generational accumulation (Figs 2 and 3, Supplementary Fig. 4a). In these lines, it is difficult to distinguish which CG-DMPs have biological significance and which do not, although one area of increasing CG-DMP concentration stretched nearly 2 Mb along chromosome 3, centred on the 10-Mb mark in all samples (Fig. 3, Supplementary Fig. 4a). CG methylation changes in this region were already apparent starting in the first generation of *msh1* −/−. The *MSH1* +/− heterozygote and all *msh1* −/− mutants showed a preference for CG hypermethylation over hypomethylation compared with the wild-type segregant, in both genes and transposons (Supplementary Fig. 4).

Similar to CG methylation, non-CG-DMPs trended towards hypermethylation in all *msh1* −/− mutants; again, methylation changes begin within the first generation and before the emergence of altered phenotype, although non-CG hypermethylation was most pronounced in the *msh1* gen2 dwarf (Fig. 2b; Supplementary Fig. 4). The vast majority of non-CG-DMPs are located in transposons around pericentromeric regions (Figs 2 and 3, Supplementary Figs 4 and 5). Within transposons, non-CG-DMPs are generally enriched around TE boundaries (Fig. 3b, Supplementary Fig. 4d). A minority of CHG-DMPs were also found in genes, with the greatest number occurring in the *msh1* gen2 dwarf samples, possibly a consequence of methylation spreading from nearby silent chromatin.

### MSH1 enhanced growth as a second methylome reprogramming

Having observed non-random methylation differences between the near-isogenic *msh1* mutants and their wild-type siblings, we performed bisulfite sequencing of two epiF_3_ individuals from an enhanced growth line and two wild-type Col-0 individuals from stock seed. From the longstanding *chm1-1* line (*msh1* advanced), two individuals with mild phenotype were selected for bisulfite sequencing. As expected, due to greater generational distance, both the *msh1* advanced and epiF_3_ lines displayed numerous genic pairwise CG-DMPs relative to wild-type Col-0 (Figs 2b and 3, Supplementary Fig. 4). However, CG-DMPs tended to be enriched around the centromere of chromosome 3 only in the epiF3 line (Fig. 2a). Whereas CG-DMPs tended to be hypermethylated in genes and transposons in *msh1* advanced plants and in epiF3 genes, a contrast was seen in epiF_3_ transposons, where CG-DMPs were hypomethylated (Supplementary Fig. 4c). Furthermore, while non-CG-DMPs in both *msh1* advanced and epiF3 tended to be hypermethylated in both genes and transposons (Supplementary Fig. 4c), the absolute number of hypermethylated CHG-DMPs in the epiF3 was much greater and similar to the number observed in the *msh1* gen2 dwarf (Fig. 2b). In addition, long stretches of CG-DMPs were present in large chromosomal blocks only in the epiF3 line (Fig. 3a).

Inducible pericentromeric CHG hypermethylation is not common in *Arabidopsis* methylation mutants^13^, crosses or natural populations^14^,^15^. EpiF3 samples also contained disproportionately high levels of hypermethylated CHH-DMPs, most located within transposons (Supplementary Figs 4b and 5). Because the *chm1-1* mutant, similar to epiF3 in generational distance from stock Col-0, did not contain these patterns, the changes were considered non-stochastic. The epiF3-enhanced growth line displays a unique pattern of CG hypomethylation and non-CG hypermethylation around transposons, suggesting a recent history of silencing release and reestablishment. Similarly, the large differentially methylated chromosomal regions in the epiF3 line imply large-scale reprogramming (Fig. 3a).

To detect discriminatory genome-wide patterns and perform multivariate analysis, we analysed the methylome based on group-wise differentially methylated regions (DMRs) between all *msh1* mutants and all wild-type samples, identified by BiSeq. 456 of 618 CG-DMRs and 3506 of 4071 CHG-DMRs mapped to transposons. *Gypsy*-like retrotransposons were heavily overrepresented in both contexts (Supplementary Fig. 6). In addition, 82.5% of DMR-associated transposons are annotated as containing a transposable element gene, a highly significant enrichment compared with all annotated transposons (Fisher’s exact test, *P*<2.2e−16). After separating transposons that contain or overlap a TE gene, these selected transposons had higher concentrations of pairwise CHG-DMPs in the *msh1* gen2 dwarf and epiF3 compared with transposons not associated with a TE gene (Supplementary Fig. 7). The epiF3 also exhibited CHH hypermethylation and CG hypomethylation in transposons containing a TE gene. These results indicate that epigenetic modulation of TE genes is likely a key consequence of *MSH1* loss.

**Figure 4:**
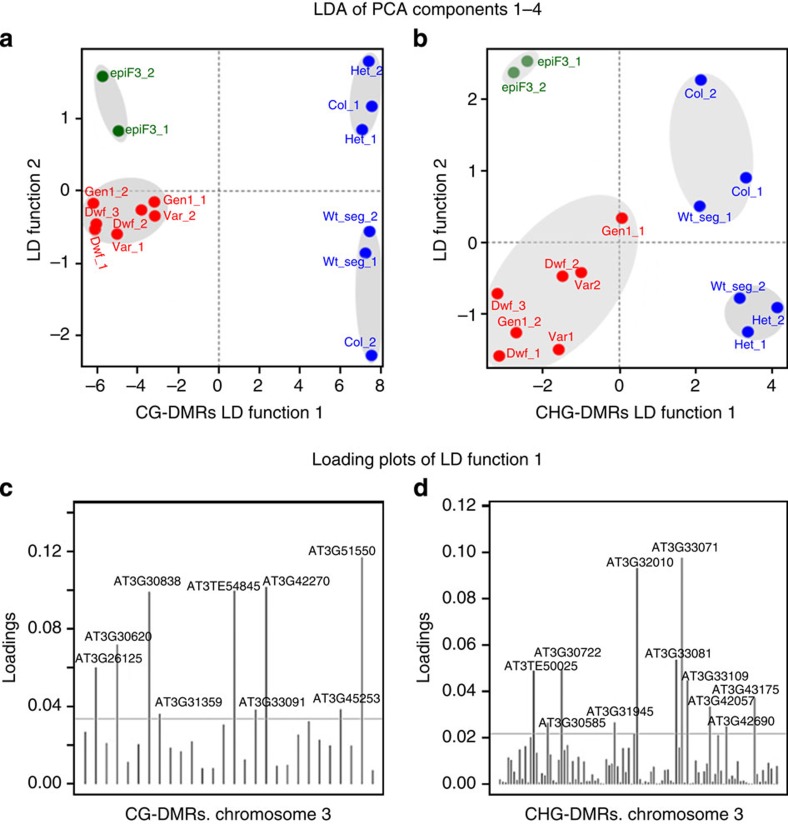
Partition of the set of samples into subsets based on genome-wide methylation patterns. (**a**,**b**) Discriminatory information conserved in two linear discriminate (LD) functions reveals the existence of genome-wide CG and CHG methylation patterns that discriminate the epiF3 lines from the subsets of mutants and wild types. (**c**,**d**) Loadings of group-wise DMRs in the LD functions indicate which DMRs have a relevant contribution in discerning between samples.

Significant genome-wide methylation differences between subsets of samples were confirmed by multivariate statistical analyses. Methylation levels in group-wise DMRs across all samples were considered as variables and reduced using principal component analysis (PCA). Subsequent application of linear discriminant analysis (LDA) revealed the existence of genome-wide CG and CHG methylation patterns able to discriminate between epiF3, *msh1* mutants and wild type (Fig. 4a,b, Supplementary Fig. 8a,b). While not all group-wise DMRs carried discriminatory information (Fig. 4c,d), signals carried by the samples were sufficient to reliably split the samples into subsets. *MSH1* +/− heterozygotes clustered with wild types, while all *msh1* −/− mutants clustered together and epiF3 samples formed a separate cluster, suggesting that epigenetic reprogramming occurs in *msh1* −/− plants and again following crossing to generate epi-lines. Multivariate analyses using methylated regions found by tiling windows were consistent with those including group-wise DMRs (Supplementary Fig. 8c–f).

### CG changes and a mobile signal in MSH1 growth effects

**Figure 5:**
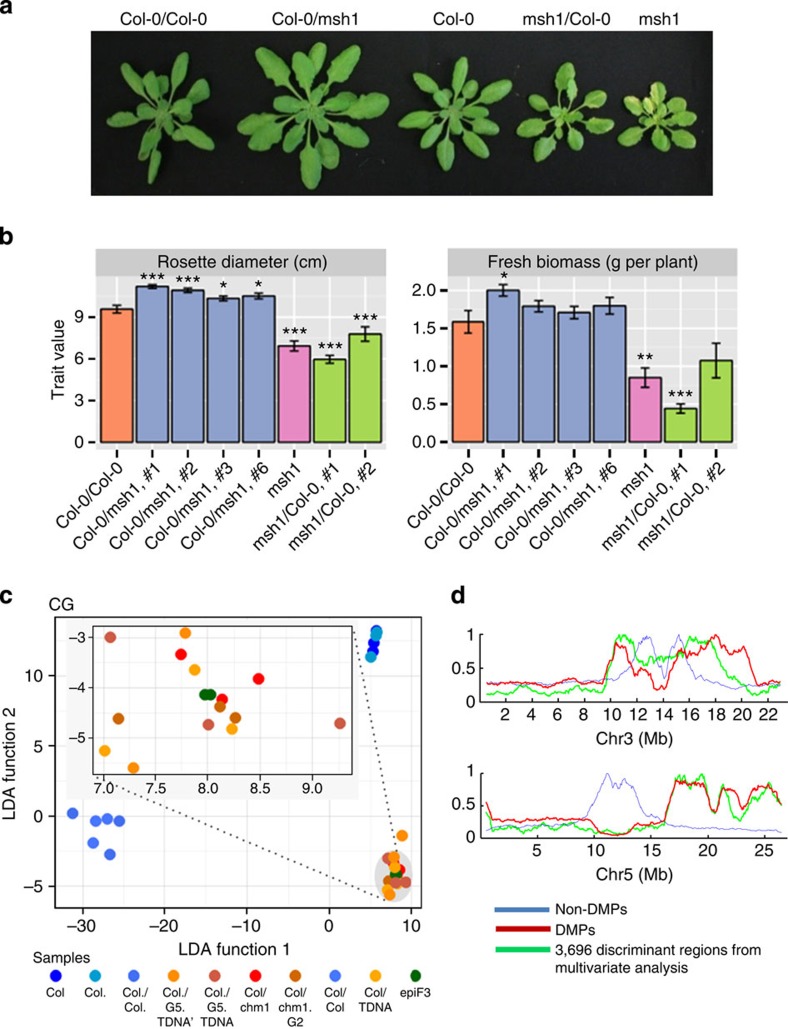
Graft transmission of the *msh1*-associated enhanced growth phenotype. (**a**) Representative plants of the first generation of progeny from grafts, designated by scion/rootstock in each case. (**b**) Rosette diameter and fresh biomass of Col-0/Col-0 control graft compared with *msh1* and the first generation of progeny from independent grafts involving Col-0 and *msh1* advanced mutant *chm1-1* (an average of 18 plants were measured; Welch’s *t*-test with *, ** and *** significant at 0.1, 0.05 and 0.001, respectively). (**c**) Three independent groups were detected by applying LDA: group 1, Col-0; group 2, Col-0/Col-0 and group 3, Col-0/chm1-1, Col-0/T-DNA and epiF3s. Pillai’s trace (1.9747) indicated highly significant statistical differences between group centroids (*P*<2.2 × 10^−16)^. Analogous results were obtained for the pairwise comparisons group 1 versus group 3 (*P*<1.7 × 10^−11^) and group 2 versus group 3 (*P*<2.2 × 10^−16^). The classification derived from LDA was independently corroborated by the consecutive application of PCA and support vector machine, which reached an accuracy of 99%. (**d**) Discriminating CG genomic regions (graft progeny from chm1-1 grafts and epiF3) overlapped with CG-DMPs for epiF3. Shown are chromosomes 3 and 5. All grafts involved floral stems and progeny measurements were taken at a single time point.

Because MSH1 downregulation produces cytosine methylation changes, we tested whether enhanced growth could be suppressed by chemical inhibition of DNA methylation or transmitted through grafting. A root growth assay showed a disproportionately greater reduction in epiF3 seedlings compared with wild type when treated with 5-azacytidine. Whereas untreated epiF3 seedlings had longer roots than untreated wild-type seedlings, 5-azacytidine-treated epiF3 seedlings were equivalent to treated wild-type seedlings (Supplementary Fig. 9), implicating some involvement of DNA methylation in the enhanced growth phenotype. When floral stem grafts between Col-0 and *msh1* mutants were generated with the mutant as the rootstock (designated Col-0/*msh1*), plants from first-generation seed had an enhanced growth phenotype reminiscent of epi-lines produced through crossing (Fig. 5a,b). This effect was not seen when *msh1* was used as scion. Progeny from graft-derived enhanced vigour plants retained growth vigour, indicating that the graft effects are heritable for at least two generations (Supplementary Fig. 10b). Similar grafting results were observed in separate experiments using *chm1-1* and *msh1* T-DNA lines (Supplementary Fig. 10a,c). Samples from both sets of graft experiments (Col-0/chm1-1 and Col-0/T-DNA) were subjected to bisulfite sequence analysis, with analysis methods similar to those used for *msh1* mutant and epiF3 samples. These experiments resulted in differential methylation patterns for first- and second-generation progeny from Col-0/chm1-1 grafts that were highly similar in behaviour to those from epi-lines derived through crossing (Fig. 5d and Supplementary Table 2 for DMP counts). In fact, LDA and clustering analysis, particularly for CG differential methylation, showed significant alignment (97% accuracy of discrimination between groups) for methylome data from both sets of grafting experiments with epiF3 data (Fig. 5c, Supplementary Fig. 10d). These observations imply that CG differential methylation is directly associated with seemingly parallel enhanced growth phenotypes derived through *msh1* crossing and grafting.

## Discussion

Data presented in this study represent the first evidence that the MSH1 effect, deriving from plastid perturbation, is accompanied by changes in epigenetic features of the genome. Previously, we showed in *Arabidopsis* that *MSH1* disruption leads to dramatically altered and heritable plant growth phenotypes^1^, and here and elsewhere^12^ have shown that crossing a plant reprogrammed by MSH1 depletion could result in striking enhancement of growth vigour. What we surmise from the present observations is that these two plant growth conditions, the initial developmental reprogramming and the subsequent enhanced growth vigour through crossing, represent two distinct methylome states of the genome. The first, characterized by variable levels of non-CG pericentromeric hypermethylation, and the second, characterized by genome-wide changes in CG methylation within the gene space.

In spite of the recent preponderance of studies related to DNA methylation, many aspects of its regulation and purpose have yet to be elucidated. However, one frequent observation is that methylation changes can occur over transposable elements, whether as a result of environmental stress^16^, or from direct disruption of the epigenetic machinery, such as in the case of *met1* and *ddm1* mutants^13^. Here we observe that manipulation of an organellar-targeted gene causes changes at similar genomic features to those found in other epigenetic studies, but with the addition of large chromosomal segments of increased epigenetic variation. One interpretation of methylation changes at transposable elements might be that as a consequence of a sufficient environmental or genomic shock, the normal methylation of transposons becomes compromised^17^. However, our observations of transposon hypermethylation rather than hypomethylation seem to argue against this explanation here.

An alternative is that, similar to suggestions of an epigenomic response to external stress, there may be feedback from the plastid to the nucleus via the epigenome. Under normal conditions, *MSH1* expression is highest in reproductive tissues^18^, and steady state transcript levels decline markedly in response to environmental stress^11^,^19^. Therefore, *MSH1* likely participates in environmental sensing via the plastid. *MSH1* downregulation triggers an altering of plant phenotype and is concomitant with epigenetic remodelling, which could be a means to relax genetic constraint on phenotype following environmental change^20^.

The increased DNA methylation variability spanning long chromosomal regions in enhanced vigour epi-lines is presumably a sign of this epigenetic remodelling, and its recapitulation through grafting implies that genome methylation and phenotypic responses are programmed. We have observed similar phenotypes from loss of *MSH1* in six different plant species^1^, further confirming that these changes are part of a preset response. Enhanced variability in growth in subsequent generations following crossing indicates that *msh1*-induced epigenetic reprogramming has special consequences when mutants are crossed to plants with unmodified epigenomes, perhaps resembling heterosis^21^,^22^. The possible role of transposons in this phenomenon requires further investigation, but studies of stress in diverse organisms suggest that there is an association between transposons, stress responses^23^,^24^ and phenotypic plasticity^25^.

## Methods

### Plant materials and growth conditions

*Arabidopsis* Col-0 and *msh1* mutant lines were obtained from the Arabidopsis Stock Center and grown at 12 h day length at 22 °C. The segregating T-DNA insertion line, SAIL_877_F01, was genotyped using forward (5′-ACGGAAAAAGTTCTTTCCAGG-3′) and reverse (5′-GCTTTCCATCGGCTAGGTTAG-3′) primers for MSH1 (At3G24320) together with SAIL primer LB3 (5′-TAGCATCTGAATTTCATAACCAATCTCGATACAC-3′). Seed from individual plants segregating for the T-DNA insertion in *MSH1* was collected from heterozygous and null *msh1* mutant plants. Progeny from a single heterozygous parent were grown to produce wild-type segregants, heterozygote segregants and first-generation *msh1* mutant segregants. Second-generation *msh1* mutants were derived from individual first-generation *msh1* mutant plants. The advanced generation *chm1-1* mutant was described previously^26^. *MSH1* first-generation, second-generation and epi-lines were derived as shown in Fig. 1. *Arabidopsis* plant measurements and leaf material used for DNA methylome analysis were conducted on 4–5-week-old plants before bolting. *Arabidopsis* flowering time was measured as date of first visible flower bud appearance. For hemicomplementation crosses, mitochondrial (AOX-MSH1) and plastid (SSU-MSH1) complemented homozygous lines were crossed to Col-0 wild-type plants. Each F1 plant was genotyped for transgene and wild-type *MSH1* allele and harvested separately. Three F2 families from AOX-MSH1 × Col-0 and two F2 families from SSU-MSH1 × Col-0 were evaluated for growth parameters. All families were grown under the same conditions, and biomass, rosette diameter and flowering time were measured. Two-tailed Student’s *t*-test was used to calculate *P* values.

### Creation of RNAi and T-DNA epi-lines

An MSH1 carboxyl-terminal fragment was cloned into pFGC1008 vector and transformed using *Agrobacterium* into Col-0 by floral dipping to obtain MSH1 RNAi lines. Plants surviving Hygromycin (35 μg ml^−1^) selection were genotyped for transgene presence using forward (5′-AAGCAACGCGTAAACTCGAC-3′) and reverse (5′-GGCGGTAAGGATCTGAGCTA-3′) primers. T1 plants were self-pollinated and *msh1* RNAi-null segregants showing the dwarf phenotype were selected for crossing. Col-0 was pollinated by *msh1* RNAi-null segregants to get F1 plants that were subsequently self-pollinated to give epi-F2 and later epiF3 lines (Supplementary Fig. 2). In addition, third-generation *msh1* T-DNA mutants coming from normal-looking generation-one and generation-two mutants were used to pollinate Col-0. Five normal-looking, five variegated and five dwarfed third-generation mutants were used in total. Derived F1 progeny were self-pollinated to produce F2 lines that were subsequently analysed for enhanced growth. A Col-0 line derived from a single Col-0 plant was used for crossing and was grown together with F1 and F2 progeny for comparison.

### Genome sequencing and SNP analysis of *msh1*

Genome sequencing was carried out at the Center for Genomics and Bioinformatics at Indiana University. The 20-nM dilutions were made for DNA samples prepared from mutant *msh1* and one epiF5 line. Preparation of single-stranded DNA used 5 μl 20 nM dilution and 5 μl 0.2 N NaOH incubated for 5 min and diluted with 990 μl Illumina HT1 Hyb buffer for 100 pM ssDNA stocks. Hundred μl of 100 pM stock, 397 μl Ht1 buffer and 3 μl PhiX 10 nM ssDNA control were loaded into the flowcell of the Illumina MiSeq and processing was according to the manufacturer’s instructions.

Raw paired-end reads (mate 1: 300 bp; mate 2: 230 bp) were quality trimmed with a Phred quality threshold of 20 and reads with a subsequent length of less than 50 bases were removed. Illumina TruSeq adapter (index 22) was trimmed (prefixed with ‘A’ user for adapter ligation), removing from the adapter match to the 3′ end of the read. A second pass of adapter trimming without the ‘A’ prefix was done to remove adapter dimers. Ambiguous bases were trimmed from the 5′ and 3′ end of reads, and those reads with more than 1% number of ambiguous bases were completely removed. A second pass of quality filtering was performed, again with bases lower than a Phred quality score of 20 being trimmed, and reads of less than 50 bases being removed. A PhiX (RefSeq: NC_001422) spike-in was removed by mapping the reads via bowtie2 (ref. 27; version 2.0.6) against the PhiX genome and filtering out any hits from the FASTQ files via a custom Perl script (available on request). The resulting FASTQ files were synchronized, such that only full mate-pairs remained, while orphans (only one mate exists) were stored in an separate file. Cutadapt^28^ (version 1.2.1) was used for the adapter removal, and the NGS-QC toolkit^29^ (version 2.3) and fastq_quality_trimmer^30^ (part of FASTX Toolkit 0.0.13.2) were used for the removal of ambiguous bases and quality filtering, respectively. The *msh1* genome was assembled using Velvet^31^ with a kmer value of 83, an insert length of 400 bases, a minimum contig length of 200 bases and the short paired (the PE reads) and a short read (the orphans) FASTQ files. The expected coverage (-exp_cov) and coverage cutoff (-cov_cutoff) were determined manually to be 25 and 8, respectively, by inspecting the initial weighted coverage of the first assembly. Resulting contigs were mapped back to Col-0 via blastn^32^ (version 2.2.26+) using an *e*-value of 10^−20^ and coverage was determined with a custom Perl script (available on request).

For the single-nucleotide polymorphism (SNP) and indel detection between *msh1* and Col-0, the PE reads were aligned against the TAIR10 reference version of the Col-0 genome sequence via the short read aligner bowtie2 using the –very-sensitive option and allowing one mismatch per seed (-N 1). Only the best alignment was reported and stored in a SAM file. The SAM file was processed via samtools mpileup^33^ (version 0.1.18) and subsequently filtered by a minimum read depth of 20, a minimum mapping quality of 30, and a minimum SNP or indel Phred quality score of 30 (*P*≤ 0.001). The SNPs and small indels were compared with supplementary data files from Lu *et al.*^34^ with custom-made Perl scripts (available on request). The *msh1* genome sequence data has been uploaded to the Short Read Archive under sample number SAMN0919714.

### Bisulfite-treated genomic library construction and sequencing

*Arabidopsis* genomic DNA (15 μg) prepared from Col-0, *msh1* (*chm1-1*) and epiF3 individual plants was sonicated to peak range 200 to 600 bp. Sonicated DNA (12 μg) was treated with Mung Bean Nuclease (New England Biolabs), phenol/chloroform extracted and ethanol precipitated. Mung Bean Nuclease-treated genomic DNA (3 μg) was end-repaired and 3′ end-adenylated with Illumina (San Diego CA) Genomic DNA Samples Prep Kit. The adenylated DNA fragment was ligated to methylation adapters (Illumina). Samples were column purified and fractionated in agarose. A fraction of 280 to 400 bp was gel purified with the QIAquick Gel Purification kit (Qiagen, Valencia, CA). Another 3 μl of Mung Bean Nuclease-treated genomic DNA was used to repeat the process, and the two fractions pooled and subjected to sodium bisulfite treatment with the MethylEasy Xceed kit (Human Genetic Signatures Pty, North Ryde, Australia). Three independent library PCR enrichments were carried out with 10 μl from total 30 μl bisulfate treated DNA as input template. The PCR reaction mixture was 10 μl DNA, 5 μl of 10 × pfuTurbo C_x_ buffer, 0.7 μl of PE1.0 primer, 0.7 μl PE2.0 primer, 0.5 μl of dNTP (25 mM), 1 μl of PfuTurbo C_x_ Hotstart DNA Polymerase (Stratagene, Santa Clara, CA) and water to total volume 50 μl. PCR parameters were 95 °C for 2 min, followed by 12 cycles of 95 °C 30 s, 65 °C 30 s and 72 °C 1 min, then 72 °C for 5 min. PCR product was column purified and equal volumes from each reaction were pooled to final concentration of 10 nM. Libraries were DNA sequenced on the Illumina Genome Analyzer II with three 36-cycle TruSeq sequencing kits v5 to read 116 nucleotides of sequence from a single end of each insert (V8 protocol). Early-generation *msh1* T-DNA insertion line methylomes were generated at the University of California Los Angeles BSCRC BioSequencing Core. All samples included for analysis represented a minimum of 50x coverage.

### Identification and annotation of pairwise DMPs

FASTQ files were aligned to the TAIR10 reference genome using Bismark^35^, which was also used to determine the methylation state of cytosines. One mismatch was allowed in the first 50 nucleotides (when the read length is 116) or 35 nucleotides (when the read length is 51, as in the case of early-generation *msh1* T-DNA insertion lines) of the read. Only reads that were uniquely mapped to a location in the genome were retained. Genomic regions with highly homologous sequences at other locations of the genome were filtered out.

Cytosines were considered for DMP identification if they were covered by four or more reads in each of the genotypes and covered by two or more reads as methylated cytosines in at least one genotype. For these cytosine positions, the number of reads indicating methylation or non-methylation for each genotype was tabulated. Fisher’s exact test was carried out for testing differential methylation between two genotypes at each position. Adjustment for multiple testing over the entire genome was done according to Storey and Tibshirani^36^ and a false discovery rate of 0.05 was used for identifying differentially methylated cytosines. Cytosines that were not identified as DMPs were considered as NDMPs. A less-stringent threshold was used for identifying differentially methylated cytosines of CHG and CHH; cytosines with a *P* value smaller than 0.05 were adjusted and a subsequent false discovery rate of 0.035 was used. Methylome sequence data have been uploaded to the Gene Expression Omnibus with accession number GSE36783.

Annotation from TAIR10 was used to determine the counts for pairwise DMPs or non-DMPs in genes, transposons, transposable element genes or other features. For plots of pairwise DMP distributions across features, the distance between each DMP and the boundary of its nearest gene and transposon was calculated. For each sample, DMP frequencies within non-overlapping 100-bp bins were computed from −2 to +2 kb relative to feature start and ends. Bin frequencies were normalized to the proportion of DMPs with mapped features having a length sufficient to cover each corresponding bin and then scaled as a proportion of the maximum bin frequency across all samples and contexts, as well as across feature types depending on comparison (genes and transposons, or transposons with and without TE genes).

### Identifying group-wise DMRs and subsequent multivariate analyses

Statistically significant CG and CHG group-wise DMRs were detected using the R-package BiSeq^37^. Each sample was represented as a vector in the *N*-dimensional space formed by the *N* means of group-wise DMR methylation levels detected in the previous step. Multivariate statistical analyses of the vector samples was performed using the R-package adegenet^38^,^39^. Partitioning of samples into subsets was performed by PCA followed by LDA. PCA was first applied to the data set to reduce its dimensionality. The four first PCA components were then used to perform the LDA. The LDA sample’s coordinates of two linear discriminant functions were used to perform the hierarchical clustering of the two-dimensional vector-samples by using the R-package cluster^40^. Ward’s^41^ minimum variance method was used as agglomerative hierarchical clustering procedure with the squared Euclidean distance. The resultant classification derived from the consecutive applications of PCA and LDA, was independently corroborated by the application of support vector machine. In all cases, the PCA step was used to reduce dimensions and to prevent any negative effect on the LDA performance caused by a possible correlation between the genomic regions. The LDA was applied to the principal components estimated in the PCA step. Alternative multivariate analyses, without relying on DMRs or DMPs, were also performed. In this case, the methylation levels in tiling windows of 340 bp with at least 20 covered cytosine sites were obtained using the R-package methylKit^42^. Next, each sample was represented as a vector in the *N*-dimensional space formed by the *N*-methylation regions and the steps of PCA, LDA and hierarchical clustering were performed. All the classification results were validated by performing 1,000 10-fold cross-validations.

### Grafting experiments

Wedge-cleft grafting was performed when primary inflorescence meristems reached 5 to 10 cm above rosettes and floral buds became visible^43^. Silicone tubing was used to secure the wedge grafts to help maintain contact between scion and rootstock. Graft junctions were further sealed with stretched parafilm to prevent desiccation. Grafted plants were kept in a mist chamber for 1–2 weeks until scions started to grow, after which plants were slowly acclimatized to normal growth conditions. Additional floral shoots were removed to promote growth of the primary grafted floral stem. Each grafted scion was harvested separately, giving rise to generation-one progeny. Single plants from generation-one progeny were allowed to self-pollinate to produce generation-two progeny.

### 5-azacytidine treatment and root length assays

Treatment with azacytidine can be used to nullify methylation effects^44^. Methylation inhibition assay was performed on wild-type Col-0 (C), an advanced epiF7 line (E), and the second-generation progeny of a Col-0/*msh1* graft (G). All seeds were bleach sterilized then sown on half-strength MS media containing 1% sucrose and 25 μl dimethylsulphoxide (untreated solvent control). Plates were placed vertically in a growth chamber maintained at 12 h daylight cycle and temperature of 22 °C. At 3 days post germination, half of the seedlings were transferred to similar half-strength MS plates containing 1% sucrose and 5-azacytidine at a final concentration of 50 μM. Ten days after moving to growth chamber, the plates were scanned and root lengths were measured using ImageJ. Three replicates were conducted for a total sample size of 18 for each line and treatment combination. Similar abolishment of enhanced epi-line root length phenotype was seen in two additional independent experiments where seedlings were directly germinated on half-strength MS media containing 30 μM 5-azacytidine or dimethylsulphoxide solvent control; total sample size was ≥39 for each line and treatment combination.

### Online content

Any additional Methods, Supplementary display items and source data are available in the online version of the paper; references unique to these sections appear only in the online paper.

## Author contributions

K.S.V. conducted the hemicomplementation and graft experiments, J.D.L. developed and analysed the early-generation *msh1* T-DNA materials and Y.-Z.X. analysed the epi-lines. J.Y., M.-R.S., R.S., J.D.L. and D.W. analysed the methylome data, M.P.A.-M. and K.S.V. developed the epi-lines, H.K. developed the RNAi lines, V.S. and M.-R.S. conducted the azacytidine experiments and J.-J.M.R. analysed and assembled the *Arabidopsis* genome sequence. The manuscript was drafted by S.A.M. and edited by all authors.

## Additional information

**Accession codes**: Methylome sequence data are deposited at GEO under accession number GSE36783. The genome sequence data has been uploaded to the Short Read Archive under sample number SAMN0919714.

**How to cite this article:** Virdi, K. S. *et al.* *Arabidopsis* MSH1 mutation alters the epigenome and produces heritable changes in plant growth. *Nat. Commun.* 6:6386 doi: 10.1038/ncomms7386 (2015).

## Supplementary Material

Supplementary Information Supplementary Figures 1-10 and Supplementary Tables 1-2.
