# Supplementary material for: Arabidopsis MSH1 mutation alters the epigenome and produces heritable changes in plant growth
Source: Nat Commun. 2015 Feb 27;6:6386. doi: 10.1038/ncomms7386 (PMC4351625; doi:10.1038/ncomms7386)
Supplement: Supplementary Information — Supplementary Figures 1-10 and Supplementary Tables 1-2. [file ncomms7386-s1.pdf]

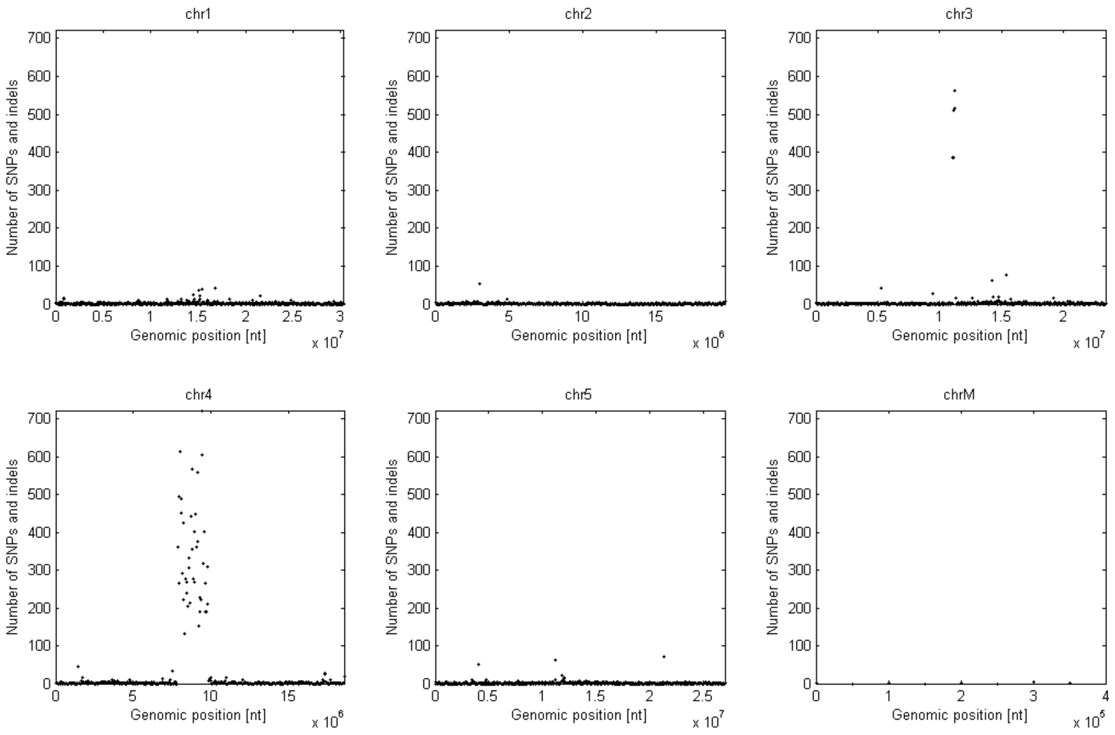

Supplementary Figure 1. Distribution of *msh1* SNPs and indels versus Col-0 across the genome. Each dot represents the number of SNPs and indels found in a window of 50kbp. Note that the Y-axis has been synchronized with the maximum number found on chr4 to enable comparisons between chromosomes. The region 7,800,000 – 9,850,0000 bp on chr4, a likely introgressed segment from Ler, contains 8582 of the total 12,771 SNPs and indels. The overlap between these data and the known SNPs and small indels of L*er* vs. Col-0 [32] is 72% and 67% for SNPs and indels, respectively. Paired-end genome-wide sequencing, alignment and *de novo* partial assembly of the chm1-1 genome produced 14,416 contigs (n50 = 40,761 bp) containing 118.5 Mbp; mapping these contigs against Col-0 covers 72Mbp. Alignment of paired-end reads to the Col-0 public reference sequence produced 95% alignment and identified 12,771 SNPs and indels, with the one 2-Mbp interval, on chromosome 4, accounting for 8,582 and the second on Chromosome 3 accounting for 2200. The *chm1-1* mutant used in this study is a Col-0 mutant once crossed to L*er*[24]*,* and the *Ler* introgressed segment on Chromosome 3 was identified genetically during positional cloning of *MSH1*[10]. Comparing SNPs and indels in the chromosome 4 interval with those in a recent study of L*er* x Col-0 [32] accounts for 5060 of 6985 SNPs (72%) and 1073 of 1597 indels (67%), consistent with a L*er* introgressed segment. Of the remaining 1988 SNP/indels , about 70% reside in non-genic regions. This SNP mutation rate appears consistent with natural SNP frequencies[14].

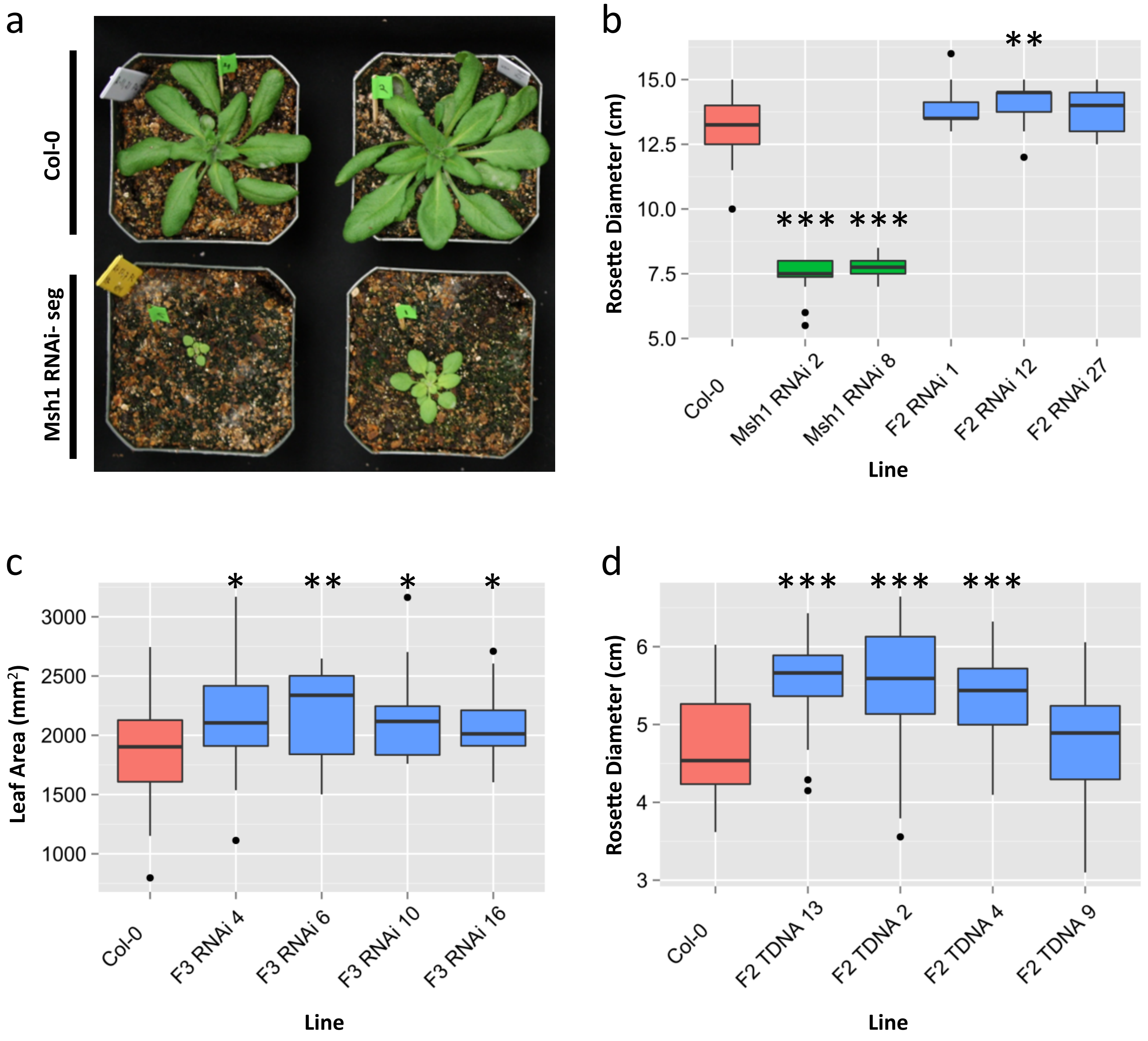

Supplementary Figure 2. Corresponding behavior in *MSH1* RNAi suppression and *msh1* T-DNA insertion lines. **a,** Null segregants (bottom row) for *msh1* RNAi transgene from self-pollinated hemizygous RNAi parents showing the dwarf phenotype. Top row, untransformed wild type Col-0. **b,** Rosette diameter of wild type Col-0, *msh1* RNAi null segregants, and F_2 plants of crosses between Col-0 and *msh1* RNAi null segregants grown at 12 hour day length. **c,** Leaf area measurements of F3 plants from selected F2 lines derived from Col-0 and msh1 RNAi null crosses. **d,** Rosette diameter of greenhouse-grown F2 plants from crosses between Col-0 and third generation msh1 T-DNA mutants. Shown are three lines exhibiting enhanced growth and one line resembling Col-0 from a total of 15 original crosses. Significance was calculated using the Wilcoxon Rank-Sum test. P-values less than 0.05(*), 0.01(**), and 0.001(***).

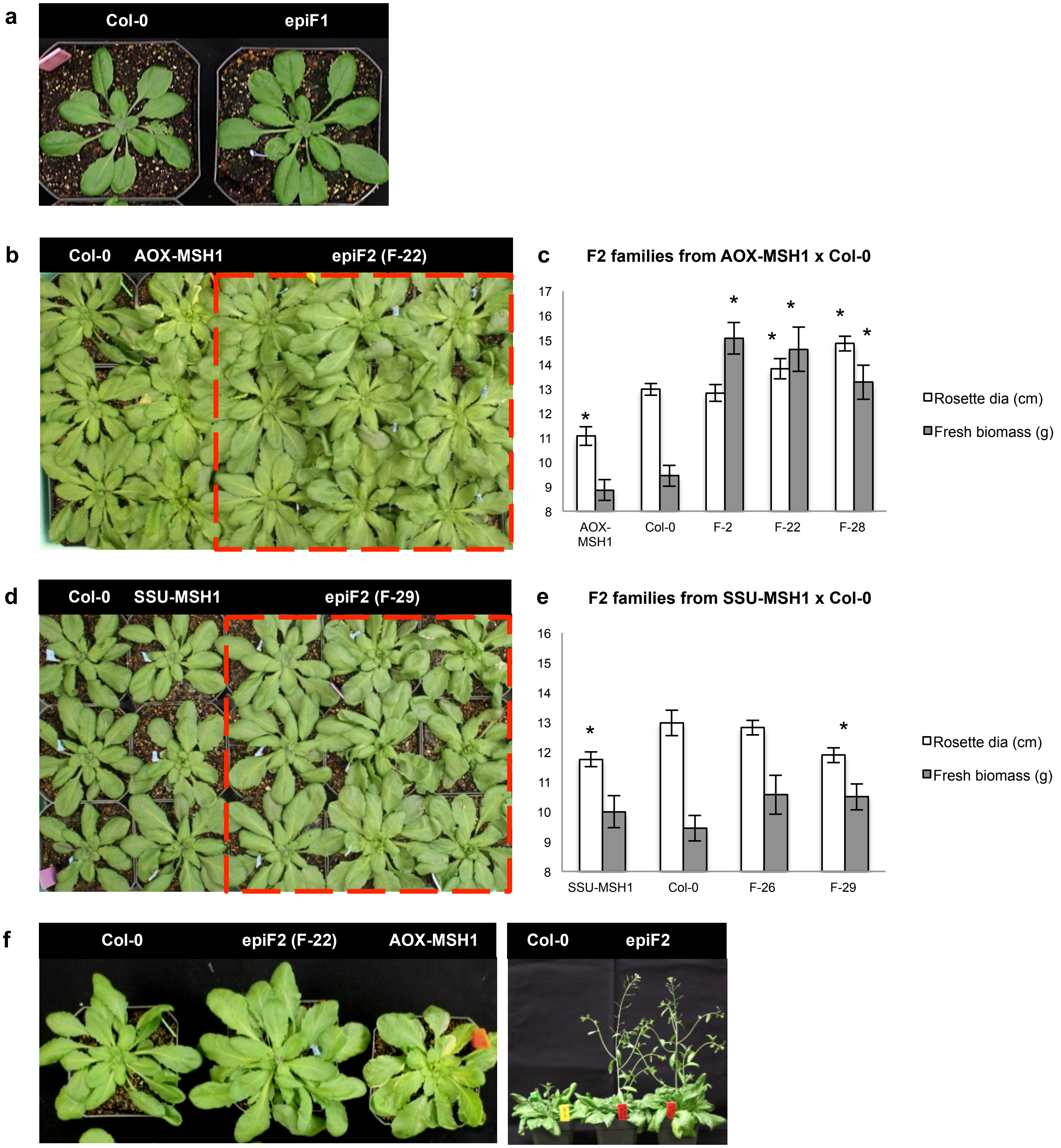

Supplementary Figure 3. MSH1-mediated enhanced growth from crossing is associated with plastid effects. **a,** Mitochondrial hemi-complementation line AOX-MSH1 x Col-0 F1. **b,** Mitochondrial-complemented AOX-MSH1 x Col-0 F2 showing enhanced growth. **c,** Rosette diameter and fresh biomass of AOX-MSH1-derived F2 lines is significantly greater than Col-0 (* $p < 0.05$). **d,** Plastid-complemented SSU-MSH1 x Col-0 F2 appears similar to wild type Col-0. **e,** Rosette diameter and fresh biomass of SSU-MSH1-derived F2 lines compared to Col-0. **f,** Enhanced growth phenotype in the F2 generation of AOX-MSH1 x Col-0.

a

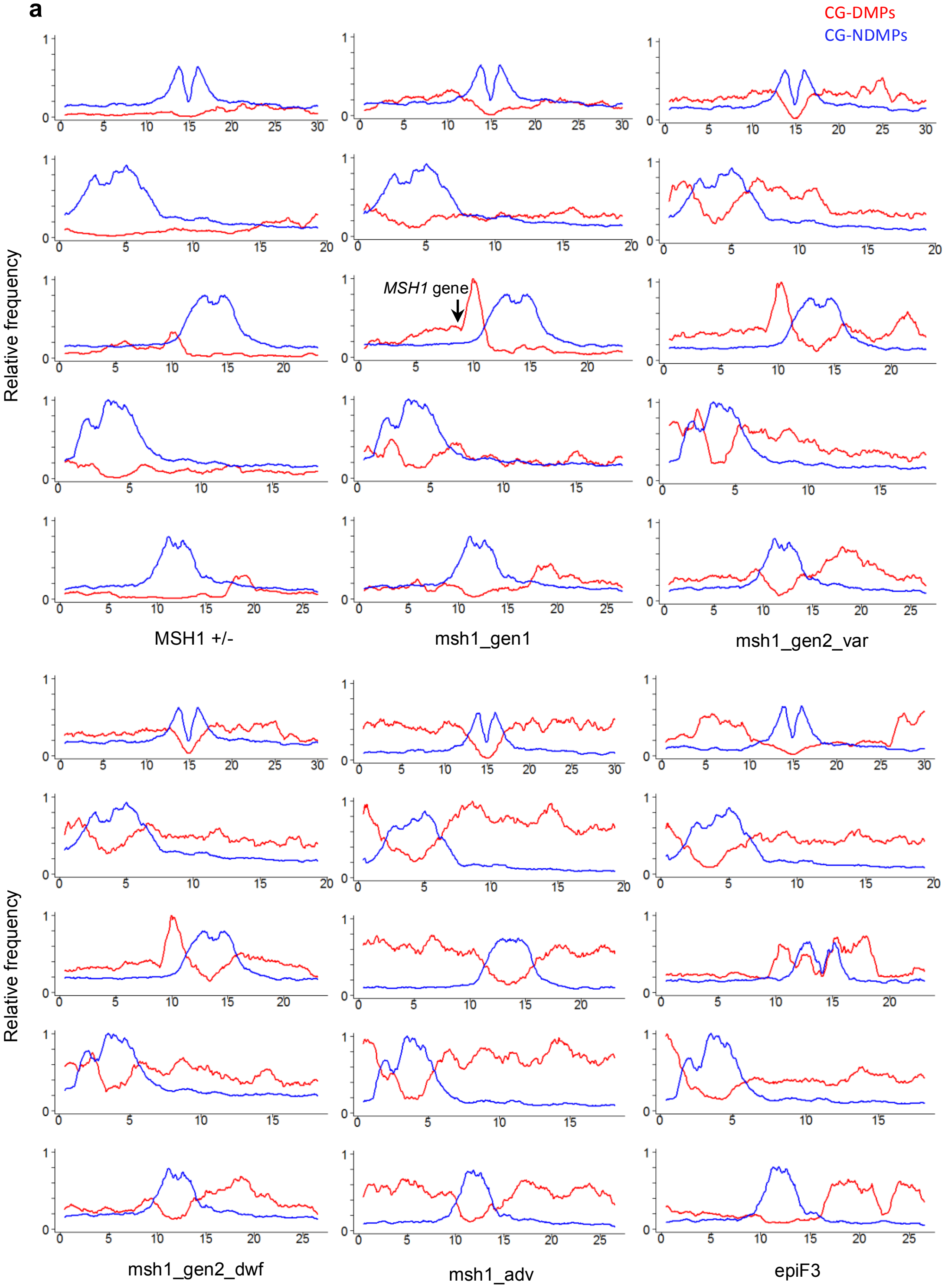
CG-DMPs
CG-NDMPs
Relative frequency
MSH1 gene
MSH1 +/-
msh1_gen1
msh1_gen2_var
Relative frequency
msh1_gen2_dwf
msh1_adv
epiF3

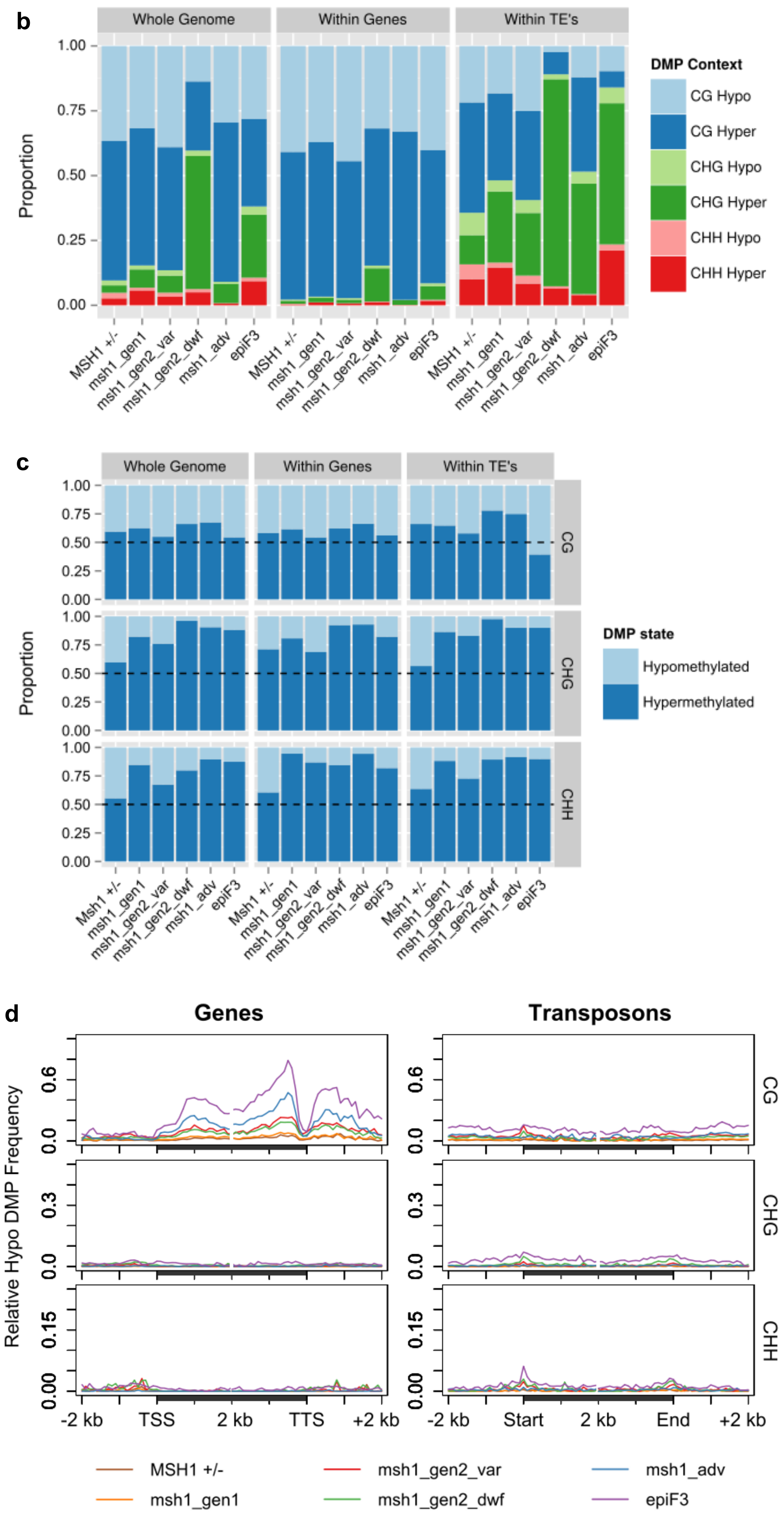

b
Whole Genome
Within Genes
Within TE's
Proportion
1.00
0.75
0.50
0.25
0.00
DMP Context
CG Hypo
CG Hyper
CHG Hypo
CHG Hyper
CHH Hypo
CHH Hyper
MSH1 +/-
msh1_gen1
msh1_gen2_var
msh1_gen2_dwf
msh1_adv
epiF3
c
Whole Genome
Within Genes
Within TE's
CG
CHG
CHH
Proportion
DMP state
Hypomethylated
Hypermethylated
Msh1 +/-
msh1_gen1
msh1_gen2_var
msh1_gen2_dwf
msh1_adv
epiF3
d
Genes
Transposons
Relative Hypo DMP Frequency
0.0
0.6
0.3
0.00
0.15
-2 kb
TSS
2 kb
TTS
+2 kb
Start
End
CG
CHG
CHH
MSH1 +/-
msh1_gen1
msh1_gen2_var
msh1_gen2_dwf
msh1_adv
epiF3

Supplementary Figure 4. Distribution of methylome changes in the msh1 and epiF3 lines. **a,** Chromosomal distributions of pair-wise CG-DMPs (red) and CG-NDMPs (blue), in a comparison of *MSH1* +/-, first generation *msh1*, second generation variegated *msh1*, and second generation dwarf *msh1* versus wild type segregant (normalized together), as well as advanced *msh1* and epiF3 versus Col-0 (normalized together). Arrow on msh1_gen1 indicates the position of the *MSH1* gene on chromosome 3. **b,** Proportion of pair-wise DMPs composed of each cytosine context within genes, transposons, and the whole genome. *msh1* second generation dwarf and epi-F3 show disproportionately high levels of CHG hypermethylation, particularly within transposons. **c,** Separate plots by cytosine context for comparison of relative hypermethylated pair-wise DMPs and hypomethylated pair-wise DMPs. *msh1* and epiF3 mutants showing higher a trend of hypermethylation, except for transposon CG methylation in epiF3. **d**, Distribution of hypomethylated pair-wise DMPs across genes and transposons, by cytosine context.

a

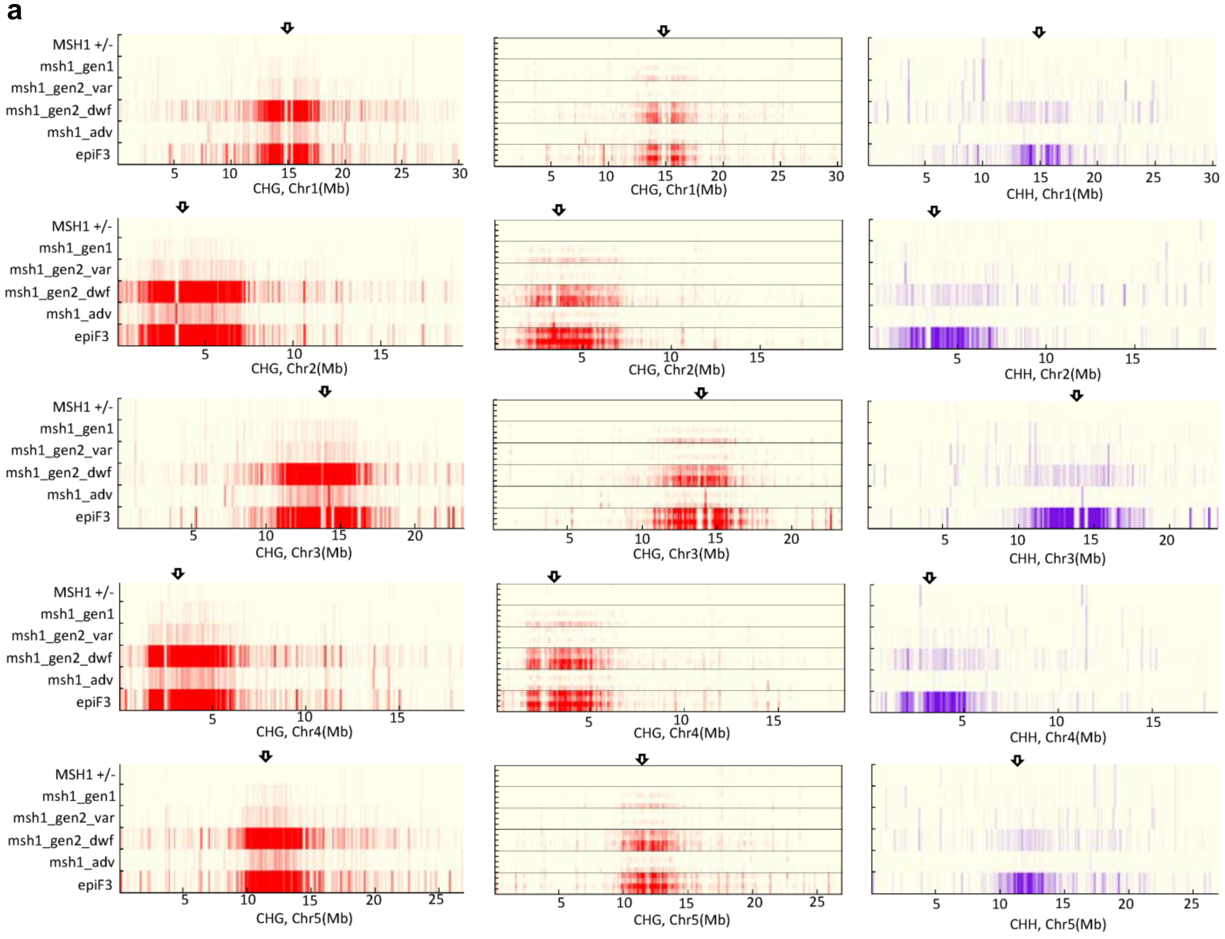

b

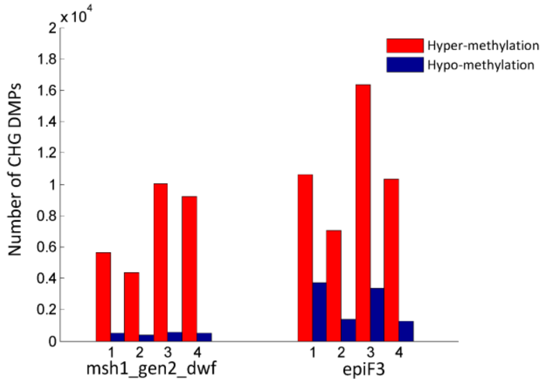

Supplementary Figure 5. Non-CG methylation changes in the *msh1* TDNA mutant and epiF3. **a,** Heat maps of pair-wise CHG-DMPs by chromosome using pooled samples (left), and individual samples with cross-comparisons in the order: mutant_rep1 vs wildtype_rep1, mutant_rep2 vs wildtype_rep1, mutant_rep1 vs wildtype_rep2, mutant_rep2 vs wildtype_rep2 (middle). Heat map of pair-wise CHH-DMPs by chromosome using pooled samples (right). Approximate location of centromere is indicated by arrows. **b,** Pair-wise CHG-DMP numbers in the cross-comparisons for msh1_gen2_dwf and epiF3, maintaining the same order as in the heat map.

a

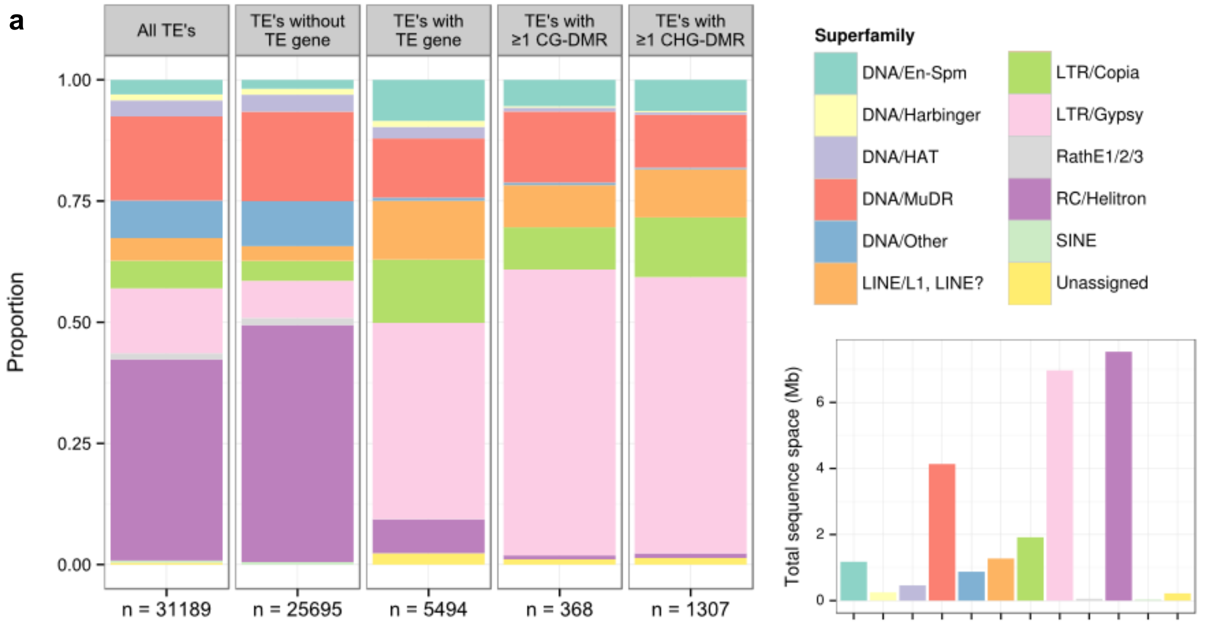

b

Superfamily	All TE's (n = 31189)	With ≥ 1 CG-DMR (n = 368 †)			With ≥ 1 CHG-DMR (n = 1307 †)		
	**Count**	**Count**	**Change**	**Adj. p-value**	**Count**	**Change**	**Adj. p-value**
DNA/En-Spm	941	20	--	--	84	--	--
DNA/Harbinger	379	1	--	--	3	**Under**	2.49e-03
DNA/HAT	1035	3	--	--	7	**Under**	2.22e-03
DNA/MuDR	5410	54	--	--	143	**Under**	4.32-e06
DNA/Other	2419	2	**Under**	6.63e-03	4	**Under**	8.44e-10
LINE/L1, LINE?	1447	32	**Over**	0.0176	130	**Over**	1.75e-08
LTR/Copia	1781	32	--	--	161	**Over**	0.0201
LTR/Gypsy	4181	217	**Over**	2.24e-23	745	**Over**	4.01e-75
RathE1/2/3	391	0	--	--	0	--	--
RC/Helitron	12945	3	**Under**	< 1.00e-75	12	**Under**	< 1.00e-75
SINE	131	0	--	--	0	--	--
Unassigned	129	4	--	--	18	--	--

† Many transposons contained more than one group-wise DMR. In total, 618 group-wise CG-DMRs and 4071 group-wise CHG-DMRs were identified.

Supplementary Figure 6. Transposon types altered in methylation. **a,** By count, *Gypsy*-like retrotransposons are highly enriched among transposons overlapping group-wise CG and CHG-DMRs in our material. To a lesser degree *LINE* and (for CHG) *Copia*-like elements are also enriched. This superfamily distribution generally resembles that of transposons which are associated with an intact transposable element gene. Bottom right: enrichment of particular superfamilies is unlikely to be an artifact from the amount of sequence space occupied by those superfamilies across the genome. **b,** Table of counts for each transposon superfamily, with Benjamini-Hochberg adjusted p-values for significantly under or over-represented superfamilies (based on Wallenius' non-central hypergeometric distribution) overlapping with group-wise DMRs. For this test, transposons were weighted by median superfamily sequence length to counter potential length bias in DMR overlap.

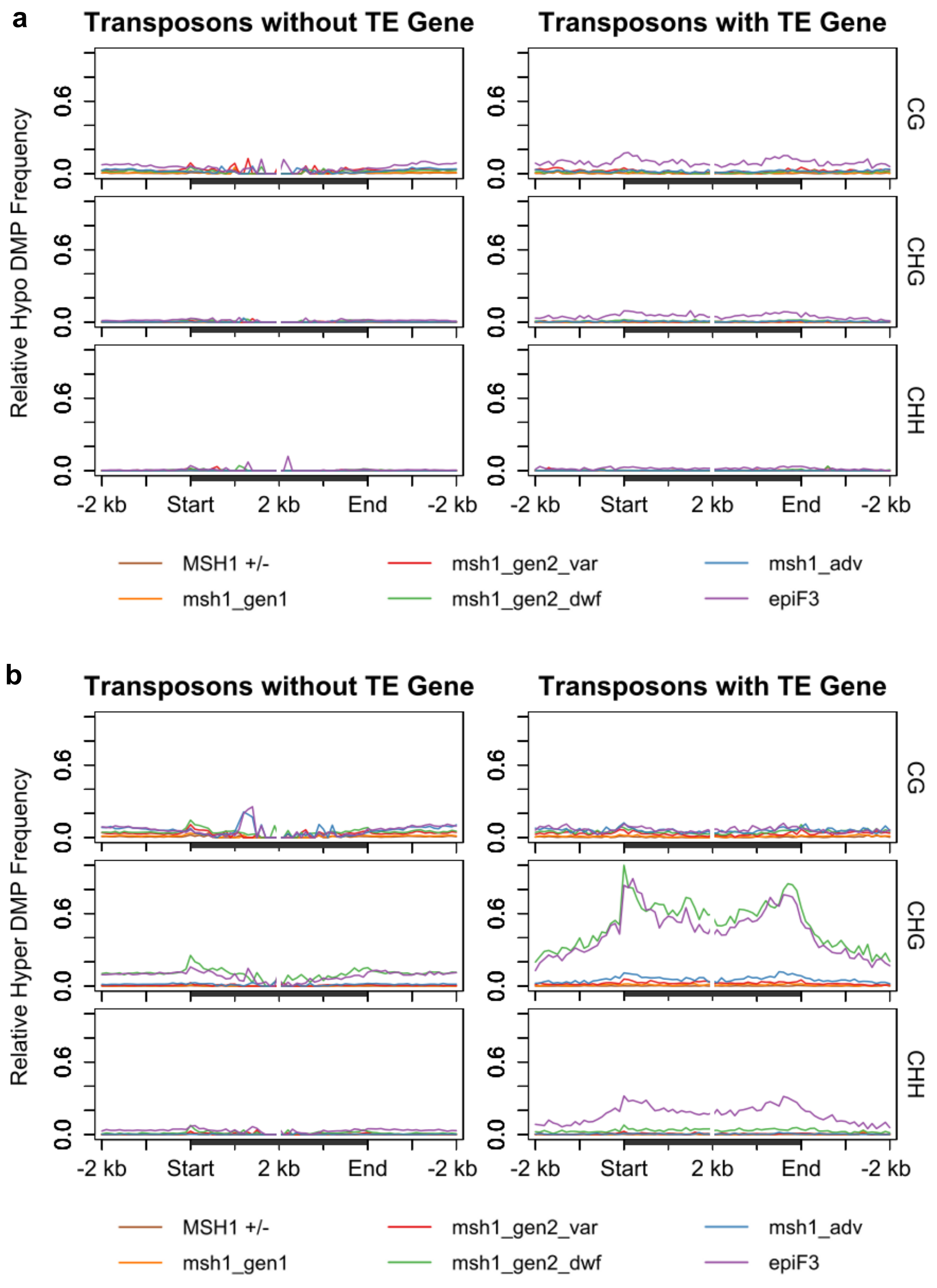

Supplementary Figure 7. Distribution of relative (**a**) hypermethylated and (**b**) hypomethylated pair-wise DMP frequencies across transposable elements that do not contain or overlap with a TE gene (n = 25695) and those that do contain or overlap with a TE gene (n = 5494), by cytosine context. Unsurprisingly, transposons not associated with a TE gene are typically shorter than those that are (median bp length = 251 and 1194, respectively; Wilcoxon test p-value $< 2.2 \times 10^{-16}$). Fluctuations in CG-DMP frequencies within bodies of transposons that are not associated with a TE gene are due to the relatively small number of such transposons that are long in sequence length.

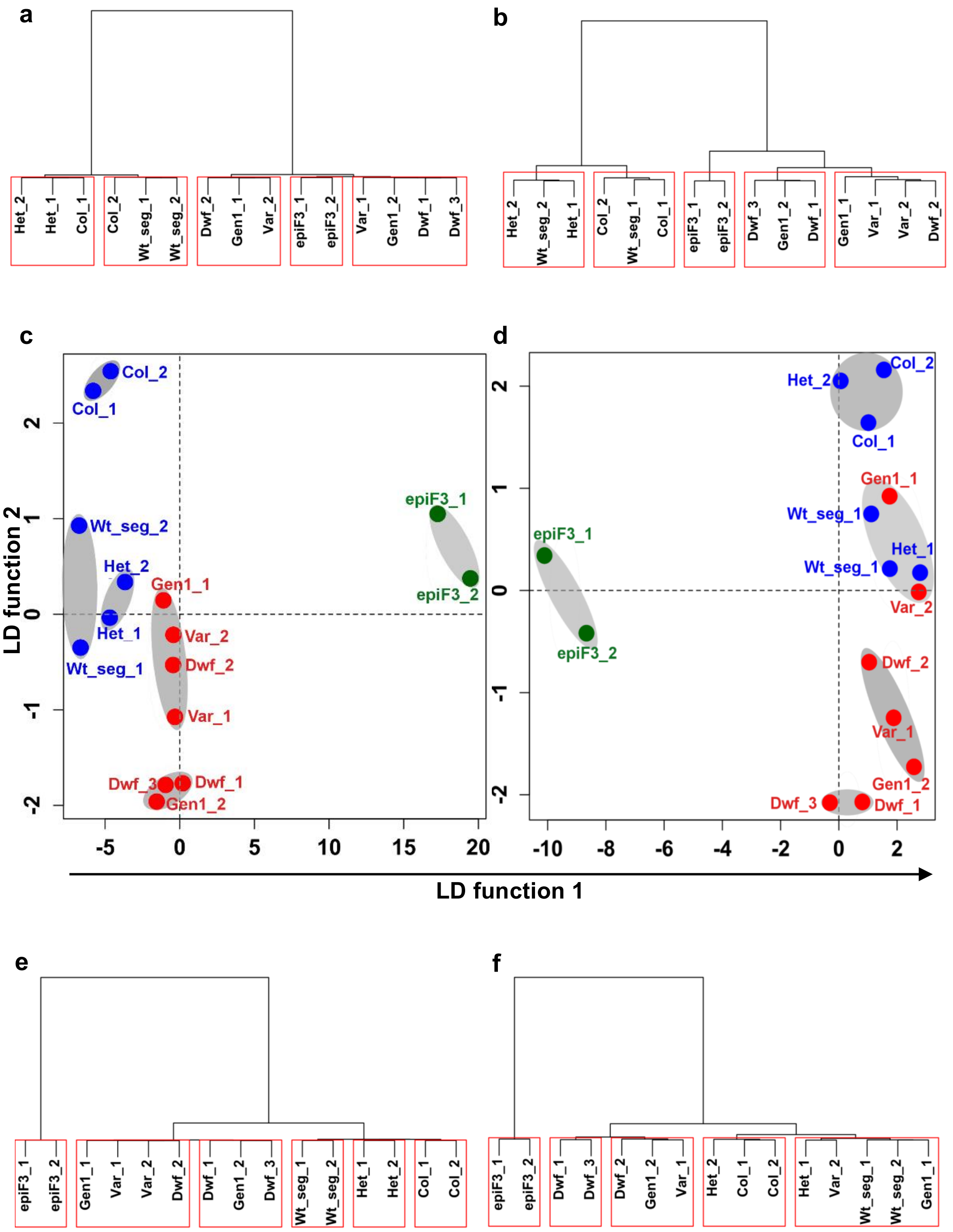

Supplementary Figure 8. Clustering based on the LDA coordinates of the samples. Hierarchical clustering for (**a**) CG and (**b**) CHG methylation of the LDA presented in Fig 3A and B of the main text, respectively. LDA for (**c**) CG and (**d**) CHG methylation regions of window size of 340 bp with at least 20 cytosine coveraged sites; panels (**e**) and (**f**) are their corresponding hierarchical clustering,

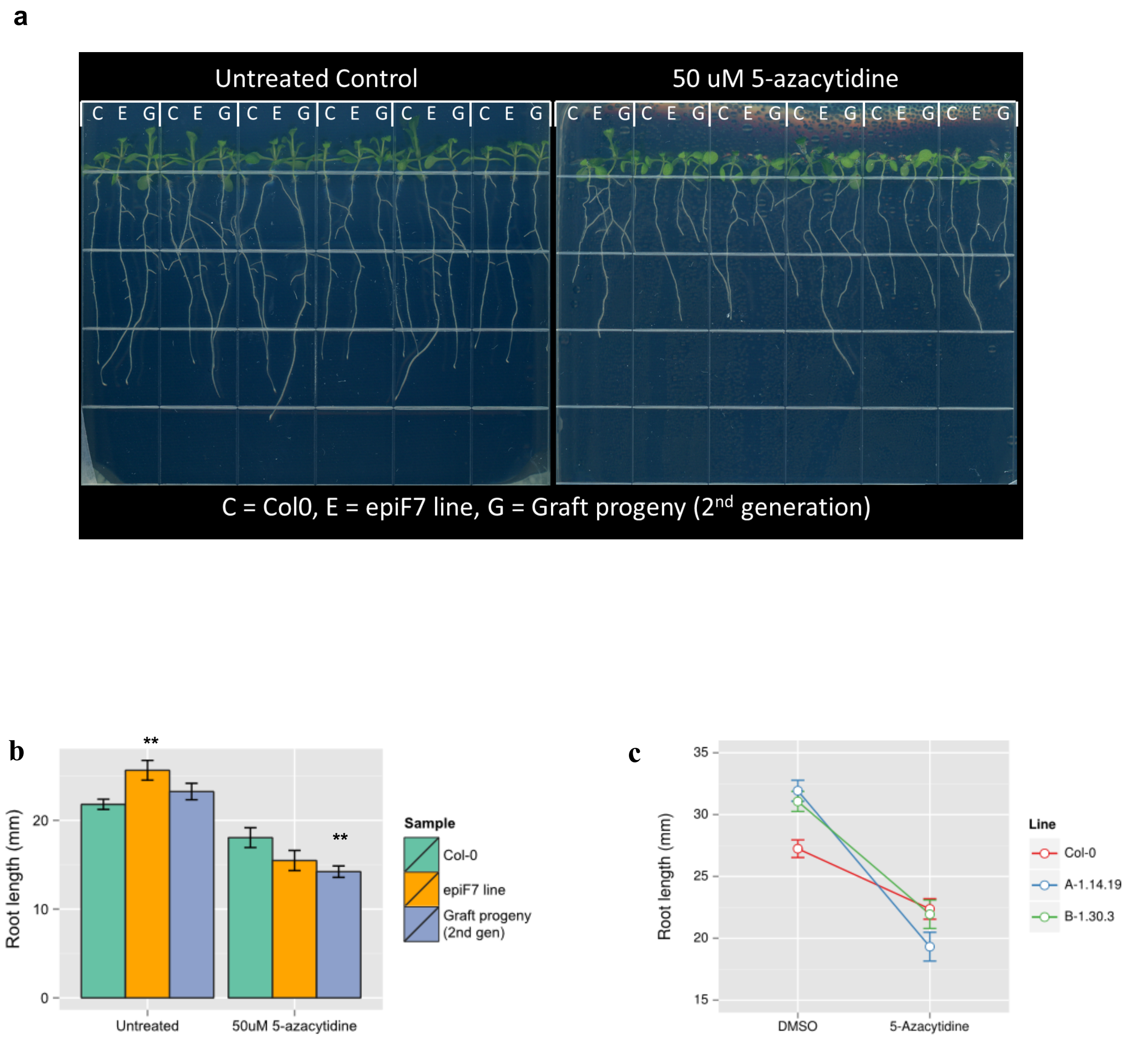

** Significant compared to Col-0 with same treatment, at $p < 0.01$.

Supplementary Figure 9. Epi-lines are disproportionately affected by 5-azacytidine treatment. **a,** For treatment of seedlings with the methylation inhibitor 5-azacytidine, seeds were alternately arranged as shown to minimize the effect of spatial variation. **b,** Increased epi-line root length is abolished by 50 µM 5-azacytidine. To assess the significance of the differences between the lines under control treatment versus 5-azacytidine, root length data was fit to the linear model $Y_{ijk} = line_i + treatment_j + (line * treatment)_{ij} + \varepsilon_{ijk}$; two-way ANOVA then indicated that the $line * treatment$ interaction term was significant (*F* = 6.60, df = 2, p-value = 0.002). **c,** An independent set of root length assays highlights the disproportional reduction of root length in epi-line seedlings. The two chosen epi-lines show signs of enhanced vigor early in development, including an approximately 20% increase in root length, which is abolished when grown in media containing 30 µM 5-azacytidine.

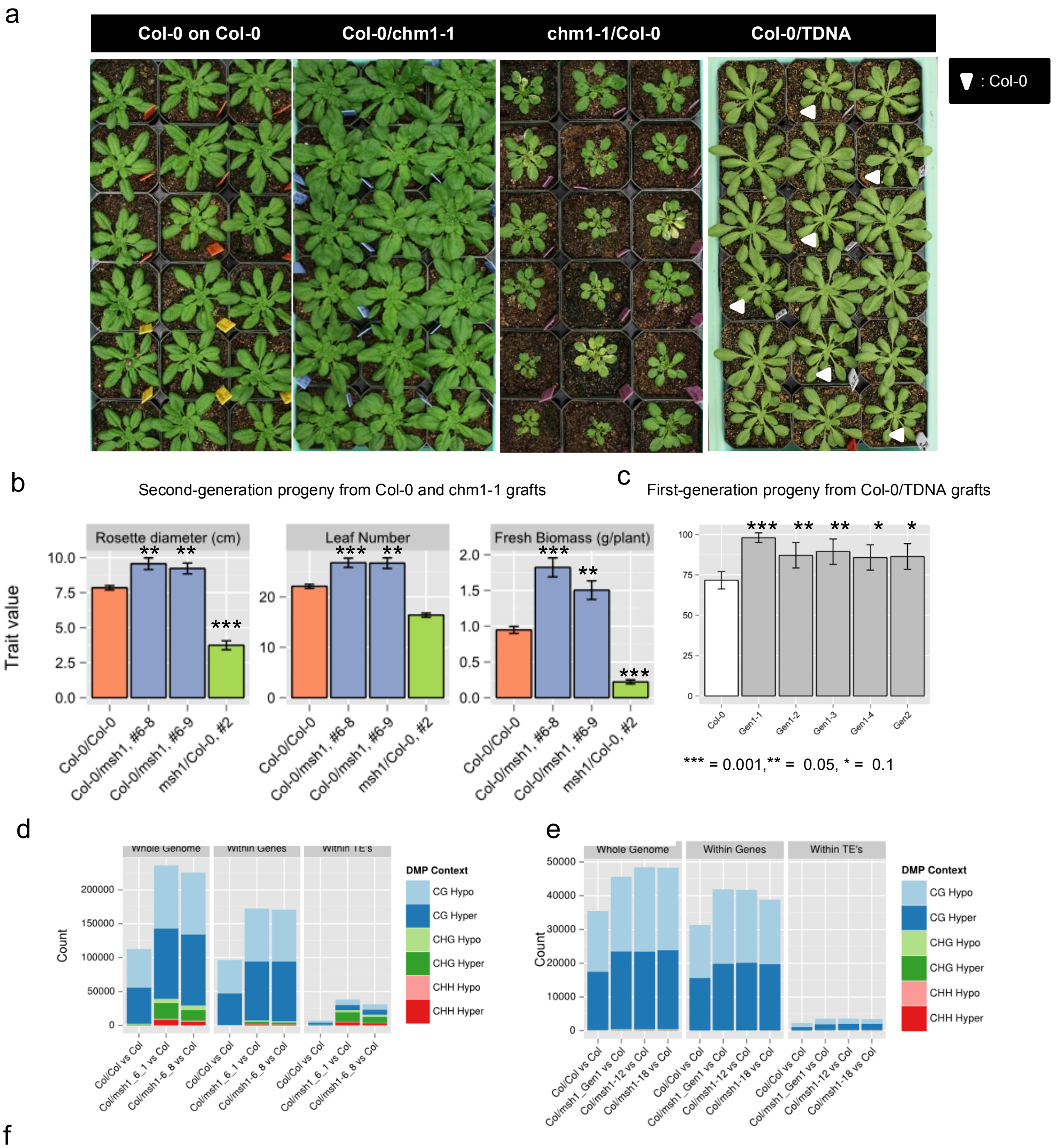

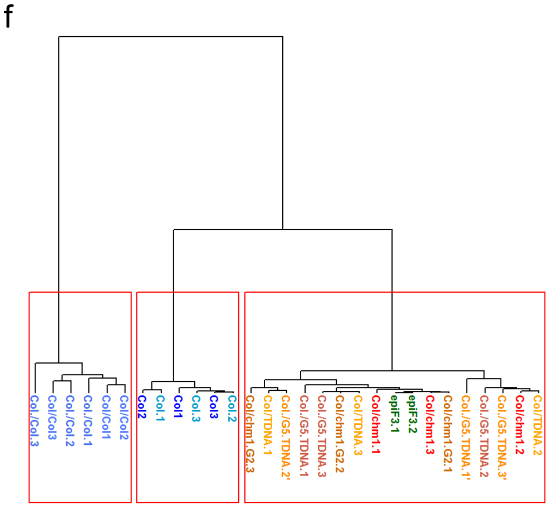

Supplementary Figure 10. Enhanced growth progeny from Col-0 scions grafted to *msh1*. **a,** First generation progeny from grafts involving both *chm1-1* and T-DNA msh1 mutant root stocks used for measurements in Figure 5b. Col-0 (white arrows) **b,** Rosette diameter, leaf number and fresh biomass of second generation progeny from grafts derived by self-pollination of first generation progeny from Col-0/chm1-1 grafts. An average of 18 plants were measured; Welch's t-test: *, **,*** significant at 0.1, 0.05, 0.001. **c**, *msh1* T-DNA influenced Col-0 scions to produce enhanced growth progeny. "Gen1" and "Gen2" indicates rootstock from first or second generation *msh1* mutants, respectively, as described in Figure 1e. **d,** Hierarchical cluster based on LDA coordinates of samples presented in Figure 5c.

Supplementary Table 1

	Rosette diameter					Fresh biomass				
	**Mean (cm)**	**N**	**S.E.M.**	**S.D.**	**p-value**	**Mean (g)**	**N**	**S.E.M.**	**S.D.**	**p-value**
AOX-MSH1	11.07	36	0.37	2.23	< 0.001	8.86	10	0.47	1.33	NS
SSU-MSH1	11.76	18	0.26	1.10	< 0.001	10.00	10	0.55	1.55	NS
Col-0	12.98	42	0.24	1.59	-	9.45	10	0.43	1.36	-
F-2 (AOX-MSH1xCol-0)	12.83	21	0.34	1.57	NS	15.07	10	0.66	2.07	< 0.001
F-22 (AOX-MSH1xCol-0)	13.82	21	0.42	1.92	< 0.10	14.62	10	0.92	2.24	< 0.001
F-28 (AOX-MSH1xCol-0)	14.85	21	0.31	1.42	< 0.001	13.27	10	0.70	1.99	< 0.001
F-26 (SSU-MSH1xCol-0)	12.82	20	0.25	1.12	NS	10.57	10	0.66	1.74	NS
F-29 (SSU-MSH1xCol-0)	11.90	21	0.27	1.25	< 0.001	10.50	10	0.45	1.19	NS

P-values are based on two-tailed Student *t*-tests compared to Col-0.
NS = not significant

Supplementary Table 2
DMP counts. In parentheses: (% of DMPs hypermethylated , % of DMPs hypomethylated)

		WHOLE GENOME		
**Sample**	**Reference**	**CG-DMPs**	**CHG-DMPs**	**CHH-DMPs**
Col-0/Col-0	Col-0	45955 (47.2%, 52.8%)	2012 (49.4% , 50.6%)	77 (36.4% , 63.6%)
Col-0/chm1 #6	Col-0	76759 (52.7% , 47.3%)	13421 (87.8% , 12.2%)	3808 (87.6% , 12.4%)
Col-0/chm1 #6-8	Col-0	77018 (53.3% , 46.7%)	9638 (82.4% , 17.6%)	2385 (80.4% , 19.6%)

		ONLY WITHIN GENES		
**Sample**	**Reference**	**CG-DMPs**	**CHG-DMPs**	**CHH-DMPs**
Col-0/Col-0	Col-0	38531 (48.4% , 51.6%)	468 (36.3% , 63.7%)	11 (27.3% , 72.7%)
Col-0/chm1 #6	Col-0	64626 (52.4% , 47.6%)	2091 (77.3% , 22.7%)	645 (82.9% , 17.1%)
Col-0/chm1 #6-8	Col-0	64830 (53.0% , 47.0%)	1634 (72.9% , 27.1%)	503 (79.3% , 20.7%)

		ONLY WITHIN TE'S		
**Sample**	**Reference**	**CG-DMPs**	**CHG-DMPs**	**CHH-DMPs**
Col-0/Col-0	Col-0	2623 (50.7% , 49.3%)	820 (72.0% , 28.0%)	19 (57.9% , 42.1%)
Col-0/chm1 #6	Col-0	5909 (54.2% , 45.8%)	8567 (92.0% , 8.0%)	2178 (90.0% , 10.0%)
Col-0/chm1 #6-8	Col-0	5902 (53.8% , 46.2%)	5802 (87.0% , 13.0%)	1208 (82.6% , 17.4%)
